# Potential use of transgenic domestic pigs expressing recombinant human erythropoietin in diabetes translation research

**DOI:** 10.1080/19768354.2018.1554544

**Published:** 2018-12-18

**Authors:** Sun-Young Baek, Hak-Jae Chung, Kyung-Woon Kim, Kyu-Ho Cho, Inchul Choi, Hoon-Taek Lee

**Affiliations:** a Swine Science Division, Rural Development Administration, Cheonan, Republic of Korea; b Division of Animal and Dairy Sciences, Chungnam National University, Daejeon, Republic of Korea; c Department of Stem Cell and Regenerative Biotechnology, Konkuk University, Seoul, Republic of Korea

**Keywords:** Diabetes mellitus, pig model, glucose, insulin, Erythropoietin

## Abstract

Recently, diabetes mellitus (DM) has shown rapid global increases with about five million deaths annually. Animal models are imperative to understand disease mechanisms and develop diagnostic, preventive, and therapeutic interventions in translational research. Rodent and mini-pig models have been established and widely used for DM research. However, domestic pig models are limited in spite of advantages such as pharmacokinetic and physiopathological availability. This study examines the potential use of domestic pigs expressing recombinant human erythropoietin (rhEPO) as disease and therapeutic response models for DM. We previously generated transgenic pigs (*n* = 16, EPO Tg) in which rhEPO was expressed and circulated in all organs. Thirty-two pigs, including 16 controls, were fed high fat (HF) diets for 42 weeks. Subsequently, blood samples for chemical and metabolic analysis were collected after fasting for 24 h and glucose loading for oral glucose tolerance tests (OGTTs). We found increased activation of the PI3 K/Akt signaling pathway under hypoxic conditions after rhEPO treatment, and HF diet-inducible-obesity in the EPO Tg and control pigs. OGTTs showed lower fasting glucose levels in the EPO Tg pigs than in controls before and after the HF diet, suggesting that rhEPO may affect glucose concentrations. Insulin and C-peptide concentrations responded slowly to glucose administration and returned to initial levels after 2 h. The blood test results suggest that EPO might affect metabolic and chemical components such as glucose, high-density lipoprotein, glucagon, triglyceride, and free fatty acid. Our findings support the use of rhEPO transgenic domestic pigs as model animals for translational DM research.

## Introduction

Diabetes mellitus (DM) is a chromic disorder associated with carbohydrate, fat, and protein metabolism, caused by absolute or relative insulin deficiency, and characterized by hyperglycemia. Traditionally, there are four main types of DM: (1) Type 1 DM is immune-mediated diabetes (also known as insulin-dependent, juvenile, or childhood-onset diabetes) caused by autoimmune destruction of the beta-cells of the pancreas, leading to absolute insulin deficiency; (2) Type 2 DM, previously called non-insulin-dependent diabetes mellitus, begins with insulin resistance commonly found in obesity, including abdominal obesity, and accounts for about 90% of people with DM globally; (3) Impaired glucose tolerance and impaired fasting glycemia are intermediate glucose levels that are above normal blood glucose values although not high enough to be considered Type 2 DM; and (4) Gestational DM occurs during pregnancy (ADA 2009; Sperling et al. 2014). The global prevalence of DM has increased over the last decade and the incidence has risen rapidly in middle – and low-income countries. The World Health Organization estimated that 422 million adults were living with diabetes in 2014, and about five million patients die of DM annually worldwide (WHO 2016; Ogurtsova et al. 2017; Cho et al. 2018).

Thus, the use of animal models in the study of DM is imperative to understand disease mechanisms, such as the molecular basis and the pathogenesis of complications, and to develop diagnostic, preventive, and therapeutic interventions in translational research. A large number of rodent models for DM research have been developed either spontaneously or by using chemical, surgical, and genetic manipulations. Type 1 DM is chemically induced by high doses of streptozoticin (STZ), alloxan, or multiple low doses of STZ. Non-obese diabetic mice, diabetes-prone biobreeding rats, Long-Evans Tokushima lean rats, Komeda diabetes-prone rats, and LEW.1AR/-iddm rats with DM caused by spontaneous autoimmunity are widely used. Akita mice with a spontaneous mutation in the insulin 2 gene are genetically induced. In Type 2 DM research, obese models are extensively used as type 2 DM is closely related to obesity. For example, monogenetic mutated mice such as Lep^ob/ob^, and Lepr^db/db^, and polygenic mutated rodents such as KK mice, Otsuka Long-Evans Tokushima fatty rats, and New Zealand obese mice. Moreover, high fat feeding can also lead to obesity, hyperinsulinemia, and altered glucose homeostasis in mouse models (reviewed by Chatzigeorgiou et al. 2009; King 2012; King and Bowe 2016).

The growth factor erythropoietin (EPO) is best known as hematopoietin because it is secreted by the kidneys in response to hypoxia and triggers erythropoiesis in the bone marrow. It is thus approved for the treatment of anemia (Li et al. 2004; Maiese 2015). However, EPO receptors are detected in other tissues, and recent findings have postulated that circulated EPO has other biological roles apart from erythropoiesis. Interestingly, dialysis patients treated with recombinant human EPO (rhEPO) show lower blood glucose levels and an improvement in insulin sensitivity. In mouse models, treatment with or expression of rhEPO reduces fasting blood glucose levels and body mass, and protects beta-cells from chemical and autoimmune toxicity, suggesting a potential antidiabetic effects of EPO (Drueke et al. 2006; Choi et al. 2010; Katz et al. 2010).

Although a large number of rodent models for DM research have been established and widely utilized, large animal models are a good complement to rodents in order to fill the translational gaps between rodent and human studies for drug development and clinical treatment (Park et al. 2013; Glastras et al. 2016; King and Bowe 2016). Particularly, the pig is a very useful and attractive model due to their organ systems, and their anatomical/physiological and pathophysiological similarity to humans. In addition, pigs have many advantages as a model for translational DM research. For example, feeding control, subcutaneous injections (for insulin treatment), reproductive efficiency such as the early onset of sexual maturity, short generation time, large litter size, and fast and affordable genetic modification techniques (Larsen and Rolin 2004; Aigner et al. 2010; Wolf et al. 2014; Perleberg et al. 2018).

In this study, we evaluate the potential therapeutic use of rhEPO and the application of the pig as a model for DM using an established line of transgenic pigs expressing rhEPO (Park et al. 2006). To do this, we examined fasting glucose, C-peptide, and insulin levels, and carried out oral glucose tolerance tests (OGTTs).

## Materials and methods

All chemicals and reagents were purchased from Sigma-Aldrich (St. Louis, MO, USA) unless otherwise stated.

### Animals and experiment design

All animal care and experimental procedures were reviewed and approved by the Institutional Animal Care and Use Committee of the National Institute of Animal Science (NIAS). The experiments were performed on Landrace (*Sus scrofa domestica*). All pigs (*n* = 32) were hosed individually in pens with a combination of concreate and plastic flooring. The pigs had free access to water via a drinking nipple and were fed according to the guidelines for breeder boars of the NIAS.

To investigate the application of a pig model for DM and the effects of EPO on DM, adult pigs were used for high fat (HF) diet-induced obesity studies. Group 1 consisted of control wild-type swine, fed a 42-week normal-chow(NC) diet (Ctrl WT-NC; *n* = 8). Group 2 consisted of control wild-type induced-obesity swine, fed a 42-week HF diet containing 20% beef tallow (Ctrl WT-HF; *n* = 8). Group 3 consisted of rhEPO transgenic swine, fed a 42-week, NC diet (EPO Tg-NC; *n* = 8). Group 4 consisted of rhEPO transgenic induced-obesity swine, fed a 42-week HF diet containing 20% beef tallow (EPO Tg-HF; *n* = 8). Body weight was measured every six week for 42 weeks.

### Western blot analysis

Liver tissue samples (100 mg) obtained from individual pigs were homogenized and lysed in NP40 lysis buffer (140 mM NaCl, 10 mM Tris (pH 7.4), 1 mM CaCl_2_, 1 mM MgCl_2_, 10% glycerol, 1% Nonidet P-40, 1 mM dithiothreitol, 0.5 mM phenylmethylsulfonyl fluoride, 2 ng/ml of aprotinin, and 10 ng/mL of leupeptin). After quantification of protein concentrations, the liver lysates (20 mg/lane) were separated by SDS-PAGE on 10% polyacrylamide gels, and electrotransferred onto polyvinylidene difluoride (PVDF) membranes (Millipore, Billerica, USA). The membranes were blocked with 5% fat-free milk and incubated with, anti-p-Akt (Ser473) and anti-beta actin (Cell Signaling Technology, Inc., Danvers, MA, USA). After extensive washes in TTBS (0.2% Tween-20), pAkt and beta actin were detected using an ECL-reagent (Amersham Pharmacia Biotech, Uppsala, Sweden) and CL-exposure films (Kodak, Perkin Elmer, Massachusetts, USA).

### Oral glucose tolerance test

Glucose tolerance tests were performed on three control wild-type (Ctrl WT; *n* = 3) and three EPO transgenic pigs (EPO Tg; *n* = 3) after an overnight fast (about 18 h). For the OGTT, the pigs were bottle-fed an oral glucose load of 2.0 g/kg body weight mixed with 20% glucose solution. Blood was collected from the ear vein before, and 5, 10, 30, 60, 90, and 120 min after glucose intake. Blood glucose levels were measured with test strips (Accu-Chek, Roche Diagnostics, Basel, Switzerland) at each time period.

### Enzyme-linked immunoassay (ELISA)

Blood samples were obtained from the control and EPO transgenic pigs, and plasma insulin and C-peptide concentrations were measured using porcine insulin or C-peptide enzyme-lined immunoassay (ELISA) kits (Mercodia, Uppsala, Sweden), respectively, according to manufacturer’s protocols. The detection limitations for insulin and C-peptide were 1.5 ug/L and 1140 pmol/L, respectively.

### Calculation of insulin sensitivity

Insulin sensitivity was evaluated using the quantitative insulin sensitivity check index (QUICKI) based on the fasting insulin and glucose levels obtained from the blood samples. The QUICKI formula is the inverse of the sum of the logarithms of fasting insulin (μU/mL) and fasting glucose (mg/dL) (Jonsson et al. 2006; Christoffersen et al. 2009).

### Blood test

Whole blood samples were taken via the catheter the morning after fasting. They were centrifugated and the porcine serum and plasma were sent to a laboratory in the Seoul Medical Science Institute (Seoul, Republic of Korea) for blood analysis including glucose, cholesterol, high-density lipoprotein (HDL), triglyceride, and hemoglobin A1c. Briefly, enzymatic assay (for cholesterol and tryglyceride), selective inhibition (for HDL and cholesterol), ACS-ACOD (for free fatty acid, FFA), glucose (Hexokinase method using glucose (Simens, USA)), and IFCC U.V method (for AST/ALT) analyses were performed using the ADVIA 2400 (Simens, USA) or Hitachi 7600–010 (Hitachi high-technologies corp., Tokyo, Japan).

### Statistical analysis

The data are presented as mean ± s.e.m. Statistical analysis was performed using Student’s *t* test or analysis of variance (ANOVA) using INSTAT3 (GraphPad Software, San Diego, CA, USA). A value of *P* < 0.05 was considered to be statistically significant unless otherwise stated.

## Results

### Potential use of a pig model for EPO and high-fat-induced obesity

**Figure 1.**
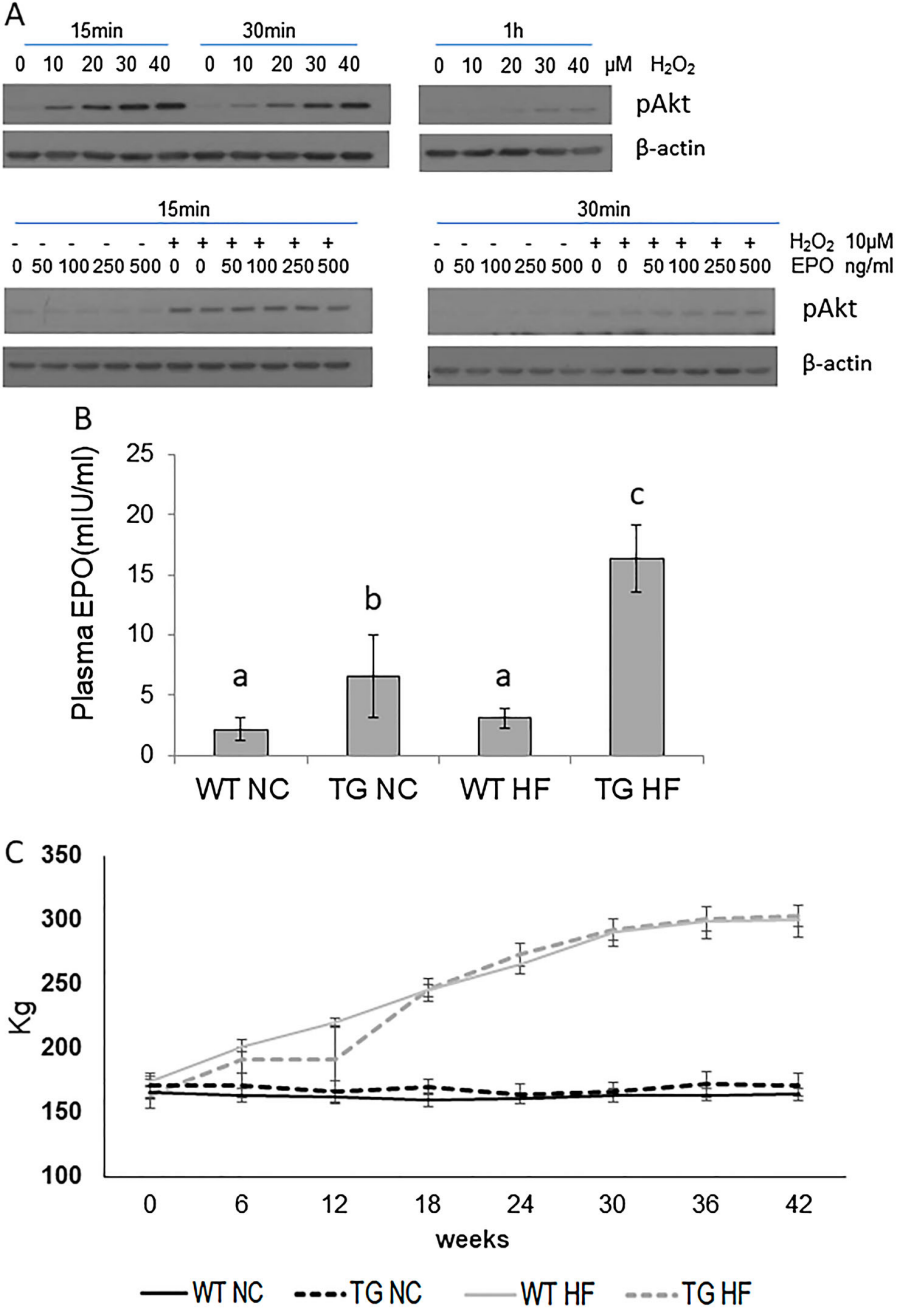
Potential use of a pig model for diabetes mellitus. A. Representative western blot analysis showing the activation of the Akt signaling pathway. B. Concentration of plasma EPO. EPO levels were measured after high fat (HF) diet or normal chow (NC) feeding. Ctrl WT NC (Control wild type pig fed normal chow), EPO TG-NC (EPO expressing transgenic pig fed normal chow), Ctrl HF (Control wild type pig fed high fat diet), EPO TG-HF (EPO expressing transgenic pig fed high fat diet). C. Body weights of high fat (HF) diet for 42 weeks. Different superscript letters differ significantly (*P* < 0.05). Values represent means ± standard error.

To determine whether rhEPO transgenic pigs can be used for DM models, we first examined whether EPO can lead to Akt (protein kinase B) phosphorylation in the liver tissues obtained from control pigs, as seen in the protective pathway of Akt and EPO in DM (Varma et al. 2005; Maiese et al. 2008). We found that pretreatment of the tissues with EPO activated hydrogen peroxide-mediated phosphorylation of Akt (Figure 1(A)).

We also measured body weight and the concentration of plasma EPO after HF or NC diets. There was no difference in the plasma concentration of EPO in the control wild-type pig groups (2.16 vs 3.1 mIU/mL). EPO transgenic pigs fed NC (6.55 mIU/mL; EPO Tg-NC) had a relatively higher concentration of EPO, than control wild-type pigs fed NC (Ctrl WT-NC; 3.1 mIU/mL)(Figure 1(B)). Interestingly, the EPO levels of transgenic pigs (EPO-Tg-HF) significantly increased after the HF diet (from 6.55 to 16.37 mIU/mL, but the HF diet did not affect the EPO level in control pigs (Figure 1(B)). The body weights of HF diet pigs, regardless of wild type or transgenic, gradually increased during the experimental period, particularly until 30 weeks (about 23 ∼25 kg/6 weeks), and the body weights of Ctrl WT-HF and EPO-Tg-HF pigs reached 303.5 ± 17.4 Kg and 299.9 ± 12.9 Kg, respectively, after a 42-week HF diet. There were no differences in relative changes in body weight between the EPO Tg-HF and Ctrl WT-HF groups after the HF diet (1.86 ± 0.06 and 1.72 ± 0.05, respectively). However, NC-fed pigs (Ctrl WT-NC and EPO Tg-NC) did not gain weight during the experiment (Figure 1(C)).

### Oral glucose tolerance test

**Figure 2.**
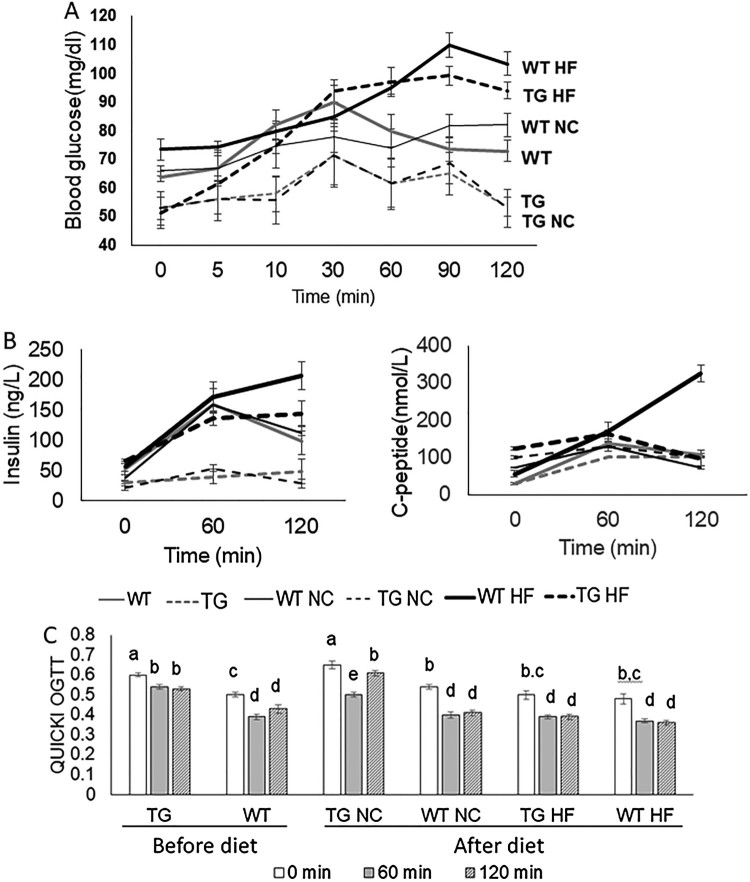
Changes in blood glucose, plasma insulin, and C-peptide. A. Changes in blood glucose before diet or after high fat (HF) or normal chow (NC) diet. B. Insulin and C-peptide changes were measured at 0, 60, and 120 min after glucose intake. C. QUICKI levels were calculated using fasting insulin and glucose concentrations. Different superscript letters differ significantly (*P* < 0.05). Values represent means ± standard error.

The fasting glucose levels in the blood plasma obtained from the EPO Tg pigs were significantly lower than those of Ctrl WT pigs before glucose loading (64 ± 1.6 vs 54 ± 3.9 mg/dL), and did not rapidly increase, compared to Ctrl WT pigs. The blood glucose level of EPO Tg pigs (53.5 ± 3.3 mg/dL) recovered to normal (initial glucose level) 120 min after glucose intake, whilst the glucose levels of WT pigs were relatively higher than initial levels before the 42-week diet experiment (HF or NC diet) (Figure 2(A)). After a 42-week NC diet, there were no significant differences in the patterns of blood glucose levels in EPO Tg pigs, in which the response to glucose loading was delayed and the highest glucose level was at 30 min post glucose intake. However a gradual increase in blood glucose concentration following glucose intake was observed in the Ctrl WT pigs. In HF diet groups, the glucose levels of EPO Tg pigs rapidly increased until 30 min after glucose loading, but the concentrations of glucose were not significantly changed and reduced from 90 min onwards. In contrast, the glucose levels of Ctrl WT pigs increased until 90 min and the levels were higher than those of EPO Tg pigs (Figure 2(A)).

### Analysis of plasma insulin and C-peptide

Plasma porcine insulin and C-peptide concentrations were measured during the OGTT. Before either the NC or HF diet, the concentration of insulin in Ctrl WT pigs was higher and the insulin response to oral glucose was fast, compared to the concentrations of insulin in EPO-Tg pigs, that did not change significantly in response to glucose loading. In a 42-week NC diet groups, similar patterns were observed. In the HF diet groups, both groups showed gradual increases in insulin levels. The patterns of C-peptide concentration in the EPO Tg and Ctrl WT pigs before the diets or after the NC diet are shown in Figure 2(B). In the HF diet groups, the concentration of C-peptide in Ctrl WT pigs gradually increased as seen in insulin responses to glucose, whilst the C-peptide concentration of EPO Tg pigs increased and returned to the initial level after 120 min.

We also evaluated insulin sensitivity using QUICKI values, and found relatively lower values in the Ctrl WT pigs, regardless of diet type, compared to EPO Tg pigs (Figure 2(C)). Moreover, Ctrl WT HF pigs had the lowest values at 0, 60, and 120 min after glucose loading, however the changes in the QUICKI patterns were not different between EPO Tg HF and Ctrl WT HF pigs.

### Blood analysis

Furthermore, we performed blood tests to provide information about overall health and compare levels between the HF and NC diet, and before and after the diet experiments (Table 1). We first checked complete blood count including red blood cells (RBC), white blood cells (WBC), hemoglobin (Hb), and hematochrit (Hct). There were no differences in WBC and RBC between the Ctrl WT and EPO Tg pigs before the HF diets, but the number of RBC was relatively lower than it was in Ctrl HF pigs. Hb and Hct were higher in EPO pigs, regardless of diet. In the metabolic panel, the overall level of examined parameters increased after the HF diet. Particularly, the levels of glucose were higher in Ctrl WT HF pigs before and after HF diets, in other words, circulating rhEPO may reduce fasting glucose levels. Interestingly, there were no significant differences in total cholesterol between the control and EPO Tg HF groups, but triglyceride levels were relatively lower in the EPO Tg groups, and more glucagon was stored in the EPO Tg HF pigs.

**Table 1.** Blood analysis.

Test	Unit	Ctrl WT	EPO	Ctrl HF	EPO Tg HF
Glucose	mg/dL	60.33 ± 5.92^a^	54.25 ± 5.13^a^	94.67 ± 4.19^b^	84.80 ± 4.59^c^
Protein	g/dL	7.65 ± 0.23	7.98 ± 0.23	8.65 ± 0.19	7.93 ± 0.19
BUN	mg/dL	11.12 ± 0.91^a^	14.05 ± 0.91^a^	6.83 ± 0.75^b^	6.96 ± 0.82^b^
AST	IU/L	28.25 ± 17.63^a^	74.50 ± 17.63^b^	31.33 ± 14.40^a^	72.83 ± 14.40^b^
GLU-FBS	mg/dL	67.50 ± 5.12^a^	55.75 ± 5.12^a^	80.67��± 4.18^b^	80.33 ± 4.18^b^
Gamma GTP	U/L	29.45 ± 5.58^a^	45.50 ± 5.58^b^	37.83 ± 4.55^c^	50.17 ± 4.55^b^
Amylase	U/L	2557 ± 332^a^	1715 ± 322^b^	3240 ± 271^c^	2460 ± 177^a^
Total Cholesterol	mg/dL	63.50 ± 7.95^a^	70.25 ± 6.65^a^	82.50 ± 6.49^b^	92.00 ± 6.94^b^
HDL	mg/dL	26.00 ± 3.04^a^	28.50 ± 4.09^a^	38.33 ± 2.51^b^	45.00 ± 2.06^c^
Potassium	mmol/L	4.85 ± 0.57^a^	6.60 ± 0.86^b^	4.69 ± 0.97^a^	6.88 ± 0.70^b^
FFA	uEq/L	141.00 ± 80.41^a^	99.50 ± 69.64^b^	297.00 ± 56.86^c^	394.83 ± 65.68^b^
HbA1C	%	22.33 ± 1.17	23.75 ± 1.01	24.17 ± 0.83	24.67 ± 0.73
WBC	Thous/uL	11.81 ± 1.37^a^	9.25 ± 1.74^a,d^	13.36 ± 1.12^b^	10.35 ± 1.21^c,d^
RBC	Mil/uL	9.06 ± 0.53^a^	9.01 ± 0.55^a^	7.39 ± 0.54^b^	9.06 ± 0.53^a^
Hb	g/dL	12.61 ± 1.48^a^	18.73 ± 1.58^b^	15.90 ± 1.21^a^	18.28 ± 1.70^b^
Hct	%	38.31 ± 4.55^a^	55.80 ± 5.52^b^	46.97 ± 3.72^c^	54.37 ± 3.18^b^
Glucagon	Pg/mL	56.33 ± 6.90^a^	47.50 ± 5.98^b^	48.33 ± 4.88^b^	61.33 ± 4.19^c^
Triglyceride	mg/dL	20.00 ± 11.22^a^	32.66 ± 9.16^b^	97.00 ± 7.95^c^	76.00 ± 7.64^d^

AST, Aspartate aminotransferase; GLU-FBS, Fasting blood sugar; Gamma GTP, Gamma glutamyl transpeptidase; HDL, High density lipoprotein; FFA, Free fatty acid; HbA1C, Glycosylated hemoglobin; WBC, White blood cell; RBC, Red blood cell; Hb, Hemoglobin; Hct, Hematocrit. Different superscript letters differ significantly (*P* < 0.05).

## Discussion

Pig models have been established for type 1 and type 2 DM and widely used to examine the effects of novel drugs on DM and DM-associated disease such as atherosclerosis, and to elucidate the metabolic mechanisms associated with hyperglycemia (Bellinger et al. 2006; Manell et al. 2014). Previous studies have reported that circulating EPO released by the liver and kidneys or the administration of rhEPO improved glucose metabolism and reduced body weight in mouse models and human patients (Choi et al. 2010; Scully et al. 2011; Zhang et al. 2017). In the present study, to demonstrate the potential use of a domesticated and rhEPO-producing transgenic pig (Landrace) as a model for DM translational research, we tested EPO responses to hypoxia and the activation of cellular signals such as the PI3K-Akt pathway (Choi et al. 2010; Cokic et al. 2014), and examined whether expressed and circulating rhEPO attenuate HF diet-induced obesity. Furthermore, we analyzed and compared complete blood count, and chemical and metabolic profiles in rhEPO-producing transgenic pigs.

We present here evidence for the activation of phosphorylated Akt by EPO pretreatment and hypoxic conditions, and observe that a HF diet induced an increase in body weight, suggesting that the pig model has the same signal pathway for EPO and cellular responses to stimulation, and that EPO transgenic pigs can be used for type 2 DM research. In contrast to mouse models, in which EPO treatment led to reduced body mass and blood glucose levels (Katz et al. 2010), rhEPO did not inhibit body weight gain, but the initial fasting blood glucose levels before OGTT were lower in HF diet pigs. The discrepancies are likely due to the relatively lower expression levels of rhEPO, about 10–12 fold higher than the control endogenous EPO in a mouse model vs about 5 fold higher than in control wild-type pigs (Chen et al. 2015).

We also observed that fasting glucose levels did not rise up to the level seen in control wild-types in EPO Tg pigs before or after HF-diets, implying mitigation of insulin resistance. Interestingly, C-peptide levels were similar between EPO Tg and WT pigs before the diets and after the NC diet, but after the HF diets, the levels of C-peptide were significantly lower after 120 min. The rapid degradation or reduction of C-peptide might be attributed to relatively lower glucose levels in the EPO Tg pigs (Ge et al. 2015). The blood analyses also confirmed that EPO Tg pigs had both DM and therapeutic features (such as high concentrations of total cholesterol, FFA, and Gamma GTP but low concentration of glucose and triglyceride, high levels of HDL), and showed direct effects from EPO, for example the amount of hemoglobin and hematocrit (Ghazanfari et al. 2010; Niu et al. 2016; Leone and Lalande 2017).

On the basis of our presented results, we conclude that domestic pigs expressing rhEPO can be used as DM disease and therapeutic models for translational DM research. Furthermore, we need to develop more precisely controlled transgenic pig models because the current rhEPO transgenic pig model does not have an on/off system, thus displayed DM disease and therapeutic characteristics caused by a HF diet and continuous EPO expression. For example, insulin generating or glucose dose–response rhEPO transgenic pigs would be useful for DM disease and clinical therapeutic studies.
